# Spatially resolved quantification of wheat kernel vitreousness using hyperspectral imaging and spectral unmixing

**DOI:** 10.3389/fpls.2026.1832288

**Published:** 2026-05-18

**Authors:** Seok Won Jeong, Young Won Kim, Yurim Kim, Kwang-Hyun Baek, Chon-Sik Kang, Jeong-Heui Lee, Youn-Il Park, Myoung-Goo Choi

**Affiliations:** 1 Department of Biological Sciences, Chungnam National University, Daejeon, Republic of Korea; 2 Wheat Research Team, National Institute of Crop and Food Sciences, Rural Development Administration (RDA), Wanju, Republic of Korea; 3 Department of Biotechnology, Yeungnam University, Gyeongsan, Gyeongbuk, Republic of Korea

**Keywords:** hyperspectral imaging, machine vision, spectral unmixing, vitreousness, wheat

## Abstract

**Introduction:**

Wheat kernel hardness, vitreousness, and creaseness are key determinants of milling performance, yet they reflect different physical scales of grain structure and are not necessarily coupled.

**Methods:**

We developed a digital phenotyping framework based on hyperspectral imaging and spectral unmixing to quantify these traits at both kernel and cultivar levels in a diverse panel of common wheat. Pixel-level spectral unmixing resolved glassy, intermediate, and mealy endosperm components within individual kernels, enabling vitreousness to be expressed as a continuous spatial index.

**Results:**

The hyperspectral-derived vitreousness index showed moderate associations with kernel protein content and the protein-to-starch ratio, consistent with variation in endosperm packing density, but weak relationships with kernel hardness and crease geometry. Kernel hardness, primarily determined by puroindoline genotype, showed limited association with bulk protein and starch composition. Crease geometry, quantified using composite indices from RGB images, captured macroscopic grain features largely independent of both hardness and vitreousness.

**Discussion:**

These results demonstrate that hardness, vitreousness, and creaseness represent complementary but largely independent dimensions of grain quality, corresponding to molecular-scale adhesion, mesoscale packing, and macroscopic geometry, respectively. The proposed framework provides a scalable, non-destructive approach for resolving intra-kernel heterogeneity, enabling improved digital phenotyping for wheat breeding and quality assessment.

## 1 Introduction

Wheat (*Triticum aestivum* L.) is one of the most important staple food crops worldwide, providing a major source of calories and protein for human diets (Shiferaw et al., 2013). Wheat kernel quality for milling and end-use is governed by several physical traits, among which kernel hardness (KH), vitreousness, and creaseness play central roles and therefore constitute key targets in wheat breeding and quality assessment (Dziki and Laskowski, 2005; Habernicht et al., 2002). Although these traits are frequently discussed together, they arise from different physical scales of grain structure and are not necessarily coupled.

KH is primarily determined by interactions between starch granules and the surrounding protein matrix and reflects the mechanical resistance of the endosperm (Huertas-García et al., 2023; Tu and Li, 2020). The major genetic determinant of KH is the *Hardness (Ha)* locus on chromosome 5D (Chantret et al., 2005), which encodes the puroindoline proteins PINA and PINB by the *Pina-D1* and *Pinb-D1* gene, respectively (Bhave and Morris, 2008; Morris, 2002; Sourdille et al., 1996). In soft wheat, wild-type PINA and PINB reduce starch–protein adhesion at the granule surface, whereas mutations or deletions in either gene increase adhesion and result in a hard grain phenotype (Bhave and Morris, 2008).

KH, however, does not behave as a simple binary trait. More than 20 *Pina* alleles and 30 *Pinb* alleles have been determined, producing a continuum of grain endosperm organization rather than discrete soft and hard classes (Chen et al., 2010; Wilkinson et al., 2008). Additional puroindoline-like genes on chromosomes 7A, 7B, and 7D (Chen et al., 2011, 2010), as well as loci on chromosomes 1B and 4B that contribute to extremely soft traits in durum wheat (Ibba et al., 2021; Wen et al., 2024), further expand the genetic architecture underlying kernel organization. The classical “lubricant” model of puroindoline action (Barlow et al., 1973; Greenwell and Schofield, 1986; Shewry, 2023) has been refined by studies showing that PINA induces γ-gliadin aggregation into large protein domains spatially separated from starch granules (Geneix et al., 2015; 2020). This organization reduces starch–protein contact in soft wheat, whereas its disruption in hard wheat leads to increased starch surface coverage and higher mechanical resistance. Despite its importance, KH alone does not fully explain variation in milling performance. Kernels with identical puroindoline genotypes and similar hardness values can differ markedly in internal structure, translucency, and flour extraction efficiency. This discrepancy arises because hardness reflects starch–protein adhesion at the molecular scale (Turnbull et al., 2003), whereas the spatial packing of the endosperm is controlled by the amount and distribution of protein deposited during grain filling (Legland et al., 2023; Turnbull et al., 2003; Yamazaki and Donelson, 1983).

Vitreousness captures this packing state of the endosperm: densely packed, protein-rich tissues transmit light and appear glassy, whereas loosely packed tissues with air-filled micropores scatter light and increase diffuse reflectance, appearing as mealy endosperm (Baasandorj et al., 2015; Dexter and Edwards, 1998; Fu et al., 2018; Dobraszczyk et al., 2002; Shahin and Symons, 2008). Consequently, vitreousness reflects endosperm porosity and microstructure rather than mechanical hardness per se. In addition to genetic background, vitreousness is strongly influenced by environmental conditions, particularly nitrogen availability during grain development (Horrobin et al., 2003; Rozbicki et al., 2015; Triboi et al., 2003; Williams et al., 2008). High nitrogen supply increases protein accumulation relative to starch, filling intergranular voids and producing a dense, translucent endosperm, whereas low nitrogen conditions result in insufficient protein deposition and the formation of air-filled pores. This environmental control underlies classical phenomena such as “yellow berry,” in which genetically hard wheats develop mealy endosperm under nitrogen limitation, while soft wheats can become vitreous under favorable conditions (Márquez et al., 2015; Padilla‐Torres et al., 2025; Troccoli et al., 2000). As a result, hardness and vitreousness, although often correlated, represent fundamentally different physical properties of the kernel.

Creaseness, referring to the depth, width and overall geometry of the ventral groove of the wheat kernel, represents a third, largely independent component of grain quality (Sun et al., 2007). This trait arises from developmental patterning of the ventral groove and pericarp during grain filling and primarily influences flour extraction efficiency by governing how effectively the endosperm can be separated from the bran (Ajiboye et al., 2015; Kong and Baik, 2016). Shallow and narrow creases facilitate higher milling yield, whereas deep or wide creases trap bran and reduce extraction efficiency (Mabille and Abecassis, 2003; Samson et al., 2005). Unlike hardness and vitreousness, creaseness reflects macroscopic grain geometry rather than endosperm microstructure, and is therefore expected to vary independently of internal protein–starch packing.

KH is routinely measured using the Single Kernel Characterization System (SKCS) (Sissons et al., 2000), while creaseness can be quantified using two-dimensional image analysis (Song et al., 2023), three-dimensional X-ray micro-computed tomography (µCT) (Huang et al., 2022; Le et al., 2019; Legland et al., 2023), or stereovision-based techniques (Sun et al., 2007). In contrast, vitreousness has traditionally been assessed by visual inspection of kernel cross-sections, a method that is subjective and prone to observer bias, particularly for piebald or partially vitreous kernels (USDA, 1997). More recently, objective alternatives based on digital imaging and visible and near-infrared (NIR) reflectance-based measurements have been introduced (Gorretta et al., 2006; Symons et al., 2003; Venora et al., 2009; Wang et al., 2002). Color-sorting devices have also been applied for classification of grain coat color and vitreousness (Brabec et al., 2010; Cha et al., 2025; Dowell, 2000; Pasikatan and Dowell, 2003), and transmissive imaging has enabled indirect assessment of internal porosity and endosperm microstructure by measuring light transmission through the intact kernel (Chichti et al., 2018). Nevertheless, vitreousness and creaseness have rarely been quantified together at the individual kernel level using fully automated, spatially resolved methods.

Recent studies have shown that kernel-averaged hyperspectral reflectance, particularly in the Vis–NIR and shortwave infrared regions, can classify wheat kernels as vitreous or non-vitreous using multivariate and machine-learning approaches (Gorretta et al., 2006; Özdoğan and Gowen, 2025; Shahin and Symons, 2008; Vermeulen et al., 2018; Zhao et al., 2023). However, because these approaches rely on spatially averaged spectra, they cannot resolve heterogeneity within individual kernels. Spectral unmixing methods treat each pixel as a mixture of spectrally distinct components (Sanjeevi and Barnsley, 2000) and therefore offers a means to address this limitation, but its application to wheat endosperm structure remains limited. While spectral unmixing has recently been applied to grain classification in other crop species (Jeong et al., 2024) and to identifying pine wilt disease-associated color changes in coniferous trees (Jeong et al., 2025), its ability to resolve localized compositional and structural heterogeneity within wheat kernels has not been systematically evaluated.

Most existing approaches rely on kernel-averaged measurements and therefore collapse spatial heterogeneity within individual kernels. As a result, partially vitreous or structurally heterogeneous grains cannot be adequately represented using conventional metrics. Here, we position this study as a digital phenotyping framework designed to resolve this limitation by providing spatially explicit, physically interpretable measurements of endosperm structure using hyperspectral spectral unmixing. In this study, we develop and validate a pixel-level spectral unmixing framework for spatially resolved, quantitative phenotyping of wheat kernel vitreousness, integrated with SKCS hardness measurement and RGB-based crease geometry analysis to characterize three complementary but physically independent dimensions of kernel quality within a single, fully script-based, non-destructive pipeline.

## 2 Materials and methods

### 2.1 Wheat cultivars and experimental design

The Korean common wheat (*Triticum aestivum* L.) cultivars collections developed and maintained by the National Institute of Crop Science (NICS), Republic of Korea, were used in this study. A total of 50 cultivars previously described by Choi et al. (2022) were grown during the 2024–2025 season and harvested in early summer 2025 under uniform agronomic conditions at the NICS experimental field. From these, 27 cultivars were selected to represent the diversity of hardness and pigmentation for digital phenotyping and chemical analysis. For hyperspectral imaging, a total of 12 intact kernels per cultivar were randomly selected and imaged prior to any destructive processing. For visual reference of vitreousness, 46 additional kernels per cultivar were manually sectioned and scored. For high-resolution crease analysis, an independent set of 24 kernels per cultivar was sectioned and imaged. These kernel sets were treated as independent biological replicates for each phenotyping modality.

### 2.2 Grain coat color classification

Grain coat color was initially classified using a standard sodium hydroxide (NaOH) color reaction test with a slight modification (Ram et al., 2002). Grains were immersed in a 5% (w/v) NaOH solution for 1 h, rinsed thoroughly with distilled water, and visually evaluated. In addition to visual classification, hyperspectral reflectance spectra were acquired from individual kernels both before and after NaOH treatment. Reflectance data were collected to enable subsequent spectral analysis of grain coat pigmentation.

### 2.3 Kernel hardness

Kernel hardness was measured using a Single Kernel Characterization System (SKCS Model 4100, Perten Instruments, Springfield, IL, USA). Measurements were performed on 300 individual kernels per sample, and mean values were used for subsequent analyses (Bettge and Morris, 2000).

### 2.4 Kernel-level protein quantification

Following image acquisition, 12 kernels per cultivar were randomly divided into two equal subsets. Six kernels were individually milled into fine flour for protein analysis. Approximately 20 mg of flour from each kernel was suspended in 1 mL of extraction buffer containing 50 mM Tris–HCl (pH 8.0), 4 M urea, 0.2% (w/v) SDS, and 20 mM dithiothreitol. Samples were sonicated on ice for 10 min and incubated at 55°C for 30 min to enhance protein solubilization. After centrifugation at 12,000 × g for 10 min, the supernatant was collected (Takahashi et al., 2019). Protein concentration was determined using the DC Protein Assay Kit (Bio-Rad Laboratories, Hercules, USA) by measuring absorbance at 750 nm, with bovine serum albumin (BSA) used as the standard, according to the manufacturer’s instruction. Protein values represent the mean ± standard deviation of six biological replicates per cultivar.

### 2.5 Kernel-level starch-derived glucose quantification

The remaining six kernels per cultivar were finely milled individually for carbohydrate analysis. Total carbohydrate content was determined by the enzymatic hydrolysis of starch (Smith and Zeeman, 2006), followed by phenol–sulfuric acid colorimetric detection (Dubois et al., 1956). Approximately 10 mg of flour from each kernel was suspended in 1 mL of 50 mM sodium phosphate buffer (pH 6.0) and hydrolyzed using α-amylase (10 U) and amyloglucosidase (10 U) at 50°C for 2 h. After centrifugation, the supernatant was diluted with phosphate buffer. For the colorimetric reaction, 100 µL of the diluted sample was mixed with 100 µL of 5% (w/v) phenol solution. Concentrated sulfuric acid (500 µL) was added slowly while maintaining samples on ice to control the exothermic reaction. The mixture was vortexed immediately and incubated in a boiling water bath (90–100°C) for 15 min. After cooling to room temperature, absorbance was measured at 490 nm. Carbohydrate concentration was calculated using a glucose standard curve and expressed as starch-derived glucose equivalents according to the manufacturer’s instruction. Values represent the mean ± standard deviation of six biological replicates per cultivar.

### 2.6 Hyperspectral image acquisition and pre-processing

Hyperspectral images were acquired using a SPECIM IQ camera (Specim, Spectral Imaging Ltd., Oulu,Finland) operating in the visible and near-infrared (VNIR) range of 400–1,000 nm. Imageacquisition followed established protocols described previously (Jeong et al., 2024). Radiometric calibration was performed using a 99% barium sulfate white reference panel. Noisy bands at both spectral extremes were removed, and all analyses were restricted to the 425–974 nm range. Spectral smoothing was applied using a Savitzky–Golay filter (window size = 10, polynomial order = 2) to reduce high-frequency noise (Beitollahi and Hosseini, 2018). RGB images were generated from hyperspectral hypercubes (**Research Data 1**) and used for external morphological characterization and as a visual reference for vitreousness assessment to ensure precise spatial co-registration between spectral and geometric features. Owing to the limited spatial resolution of the hyperspectral sensor (512 × 512 pixels), hyperspectral images were not used for detailed assessment of crease geometry. Prior to spectral unmixing, strict background filtering was applied to hyperspectral images to isolate valid kernel pixels. Pixels containing Not-a-Number (NaN) or infinite (Inf) values were excluded. In addition, pixels with near-zero spectral intensity (sum of absolute reflectance values across all wavelengths ≤ 1 × 10^−12^) were classified as background or shadow and removed. Only validated foreground pixels were retained for subsequent analysis.

### 2.7 Morphological characterization and visual vitreousness assessment

External grain morphology parameters included grain length (GL), grain width (GW), grain perimeter (GP), grain area (GA), aspect ratio (AR), and grain circularity (GC) (Braadbaart and Van Bergen, 2005; Choi et al., 2022). These traits were quantified from RGB images derived from hyperspectral hypercubes to ensure spatial co-registration with spectral information. For internal structural characterization related to vitreousness, kernels were manually sectioned transversely and cross-sectional RGB images generated from hyperspectral images were analyzed to quantify grain thickness (GT) and grain cross-sectional area (SA).

All morphological variables were standardized using Z-score normalization:

*Z_ij_ = (x_ij_ - μ_j_)/σ_j._*

where *x_ij_* is the value of trait *j* for cultivar *i*, and *μ_j_* and *σ_j_* denote the mean and standard deviation of trait *j*, respectively. This normalization ensured equal weighting of variables in subsequent multivariate analyses.

For visual reference measurements, vitreousness was quantified at the kernel level rather than the pixel level. For each cultivar, four independent sets of 42 transversely sectioned kernels were prepared and classified by three trained evaluators into glassy (> 70% vitreous), intermediate (30–70%), or mealy (< 30%) categories based on the proportion of translucent endosperm. Visual scoring was conducted following established standards (ICC, 1980; ISO, 1994). Visual vitreousness was calculated using the same index formulation as defined in Section 2.8, with *N_g_*, *N_i_*, and *N_m_* representing the number of kernels classified as glassy, intermediate, and mealy, respectively, and *N_total_* representing the total number of kernels (42), rather than pixel counts. For each cultivar, vitreousness values were averaged across evaluators and replicate sets, and variability was expressed as mean ± standard deviation.

### 2.8 Endmember extraction and spectral unmixing for vitreousness assessment

Hyperspectral unmixing was performed to resolve the spatial distribution of endosperm components within individual kernels using a Linear Mixing Model (LMM) with Non-Negative Least Squares (NNLS) optimization. This approach assumes that the observed reflectance spectrum of each pixel can be approximated as a linear combination of constituent endmember spectra, which is appropriate for reflectance-based measurements of heterogeneous biological tissues. Endmembers were extracted using the Pixel Purity Index (PPI) algorithm (Boardman et al., 1995) following Principal Component Analysis (PCA)-based dimensionality reduction. An initial set of 10 global endmembers was selected to capture the dominant spectral variability across the dataset while retaining minor but biologically relevant components. Endmembers corresponding to glassy, intermediate, and mealy endosperm were identified based on their spectral characteristics and spatial abundance patterns. Pixel-wise abundance fractions were estimated using NNLS under non-negativity constraints and subsequently normalized to satisfy the sum-to-one condition. Based on abundance classification, a kernel-level vitreousness index (*V*) was calculated as:

*V = (N_g_ + 0.5 N_i_)/N_total._*

where *N_g_*, *N_i_*, and *N_m_* denotes the number of pixels classified as glassy, intermediate, and mealy endosperm, respectively, and *N_total_* represents the total number of valid kernel pixels after background removal. The intermediate class was assigned a weight of 0.5 to reflect its transitional structural state. To assess robustness, alternative endmember extraction algorithms N-FINDR (Chang and Plaza, 2006) and FIPPI (Winter, 1999) were evaluated, and reconstruction error (rRMSE) was compared (**Supplementary Table S1**). The resulting vitreousness index was stable across methods. Validation was performed by comparing hyperspectral-derived vitreousness with independent visual scoring using linear regression at the cultivar level and confusion matrix analysis at the kernel level.

### 2.9 Creaseness assessment

Creaseness was evaluated independently of hyperspectral analysis because the spatial resolutionof hyperspectral images (512 × 512 pixels) was insufficient to resolve fine-scale creasegeometry. Instead, transverse kernel sections were imaged using high-resolution RGB photography(4,000 × 4,000 pixels) under controlled lighting conditions (**Research Data 3**). Crease geometry was quantified using fully automated Python-based image analysis (**Supplementary Code S1**). Kernel cross sections were first segmented to obtain the kernel outline, after which aconvex hull was constructed. Crease area (CA) was defined as the region between the convex hull andthe kernel boundary within the ventral groove. Crease width (CW) was calculated as the maximum distance across the crease opening along the convex hull boundary, and crease depth (CD) as the maximum perpendicular distance from the opening line to the deepest point of the indentation. Grain thickness (GT) and grain cross-sectional area (SA) were measured from the same segmented cross sections. To minimize size effects, dimensionless indices were computed, including CD/GT, CW/GW, and CA/SA. Creaseness was treated as a continuous structural trait; categorical classes (shallow, intermediate, pronounced) were used only for visualization within the cultivar panel. Using the same high-resolution RGB cross-sectional images, CD, CW, and GT were independently measured in ImageJ and via the automated Python pipeline to validate the accuracy and consistency of the script-based creaseness extraction (**Supplementary Code S1**).

### 2.10 Vitreousness and creaseness prediction modeling

Random Forest regression was used to predict kernel vitreousness, grain coat color, and other target traits from image-derived features. Feature vectors consisted of hyperspectral endmember abundances (for vitreousness) or crease geometry indices (for creaseness). A consistent model configuration was used across all prediction tasks to ensure comparability. The number of trees (n_estimators) was set to 100, the maximum tree depth was not restricted (default setting), and the random_state was fixed at 42 for reproducibility.

For vitreousness prediction, model outputs were inverted (*ŷ_inv_* = 1 − *ŷ*) because higher abundances of specific endmembers corresponded to mealy tissue. Predicted values were subsequently normalized using min–max scaling (*y_min_* = 0.1, *y_max_* = 0.9) to align predictions with increasing vitreousness. Model performance was evaluated using 5-fold cross-validation. To reduce the influence of extreme outliers, a two-step training strategy was applied: an initial model was fitted, and the top 5% of samples with the largest absolute prediction errors were excluded. The final model was then trained on the filtered dataset. Model performance was assessed using the coefficient of determination (R^2^), and feature importance scores were calculated to quantify the contribution of individual variables. For vitreousness, normalized predictions were further assigned to three classes (mealy, intermediate, glassy), and confusion matrices were constructed against visual reference labels.

### 2.11 Multivariate statistical analysis

Spectral data were preprocessed using Standard Normal Variate (SNV) transformation to reduce multiplicative scatter effects (Barnes et al., 1989), followed by mean-centering and variance scaling. Partial Least Squares Regression (PLSR) was used to model relationships between spectral data and target variables. The number of latent variables was selected by 10-fold cross-validation based on the coefficient of determination (R^2^) and root mean square error (RMSE). Variable Importance in Projection (VIP) scores were computed to identify wavelength regions contributing most strongly to trait prediction (Wold et al., 2001). PLSR was used primarily for spectral interpretation and variable selection, whereas Random Forest models were used for predictive modeling.

Hyperspectral, morphological, and chemical measurements were obtained from independent but cultivar-matched subsets of kernels. Because protein and starch analyses required destructive sampling, these measurements were not paired at the individual kernel level. Accordingly, associations between hyperspectral features and biochemical composition were evaluated at the cultivar level. Kernel counts per cultivar and per imaging batch are summarized in **Supplementary Table S2**, while the processed kernel-level and cultivar-level feature dataset used for statistical analyses is provided in **Supplementary Table S3**.

All linear regressions were performed using ordinary least squares (OLS), with R^2^ values reported alongside 95% confidence intervals. Group comparisons were conducted using one-way ANOVA, with effect sizes reported as η^2^ and *post hoc* comparisons performed using Tukey’s HSD test.

## 3 Results

### 3.1 Spectral, morphological, and compositional characteristics of the wheat panel

#### 3.1.1 Grain coat color and hyperspectral reflectance characteristics

**Figure 1:**
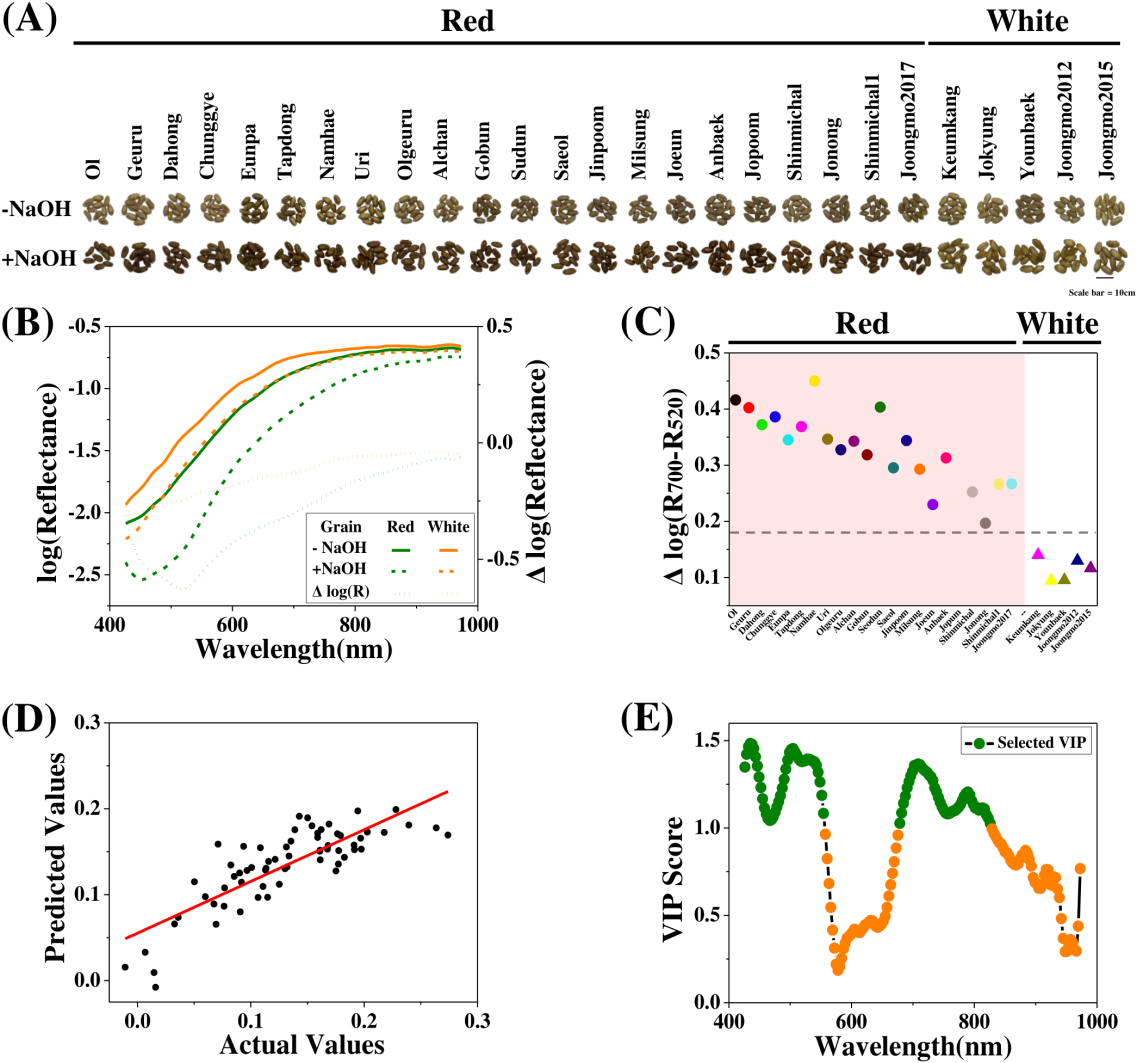
Hyperspectral discrimination of wheat grain coat color. **(A)** RGB images of representative red and white wheat kernels before (–NaOH) and after (+NaOH) sodium hydroxide treatment. **(B)** Mean log-transformed reflectance spectra of red and white kernels before and after NaOH treatment, together with difference spectra (ΔlogR = logR_−NaOH_ − logR_+NaOH_). Spectra represent cultivar-averaged means derived from 22 red and 5 white cultivars, each measured from 10 individual kernels. **(C)** Distribution of the spectral color index Δlog(R_700_ − R_520_) for each cultivar. Each symbol represents the mean value of 10 kernels from one cultivar; the dashed horizontal line indicates the classification threshold between red and white kernels. **(D)** Regression performance of the hyperspectral color model, shown as predicted versus measured Δlog(R700 − R520) values for individual kernels. Model performance is summarized by the coefficient of determination (R^2^) and root mean square error (RMSE) (R^2^ = 0.67; RMSE = 0.03). **(E)** Variable importance in projection (VIP) scores from partial least squares regression highlighting wavelength regions contributing to grain coat color discrimination. Points represents cultivar-level average; green markers indicate wavelengths selected for index construction.

To contextualize hyperspectral vitreousness phenotyping, baseline variation in grain coat color, morphology, kernel hardness, and biochemical composition was first characterized across the wheat cultivar panel. The Korean common wheat cultivar collection consists of approximately a 3:1 ratio of red to white wheats (Choi et al., 2022). From this collection, 27 cultivars were selected to represent grain coat color diversity, comprising 22 red and 5 white wheat cultivars, maintaining an approximately 4:1 ratio (**Figure 1A**). Conventional classification of wheat grain coat color relies on visual inspection following NaOH treatment (Choi et al., 2022) or reflectance measurements after NaOH soaking (Patel et al., 2016; Ram et al., 2002).

To evaluate whether hyperspectral imaging could support grain coat color discrimination and identify underlying spectral features, reflectance spectra were acquired from individual kernels both before (−NaOH) and after (+NaOH) sodium hydroxide treatment. Red kernels exhibited a pronounced decrease in mean log-transformed reflectance after NaOH treatment compared with white kernels, particularly within the visible wavelength range, whereas differences in the near-infrared region were relatively small (**Figure 1B**). This contrast became clearer when post-treatment spectra were subtracted from pre-treatment spectra (ΔlogR). The largest separation between red and white kernels was consistently observed around 520 nm, corresponding to the wavelength region of maximal ΔlogR contrast, while a secondary but stable difference was observed near 700 nm, where spectral variation was less affected by baseline intensity differences.

Based on these patterns, a simple color index, Δlog(R700 − R520), was constructed to capture both the primary contrast (520 nm) and a reference region (700 nm) to enhance robustness against overall reflectance variation. This index was calculated for individual cultivars (**Figure 1C**) with white cultivars consistently exhibiting lower index values (below approximately 0.175) and red cultivars showing substantially higher values, indicating effective discrimination between the two grain coat types. When Δlog(R700 − R520) values derived from kernels prior to NaOH treatment were used to train a random forest regression model for grain coat color classification, the model achieved an R^2^ of 0.67 (95% CI: 0.52 – 0.79; **Figure 1D**), supporting the predictive utility of this index. Residual variation was primarily observed within cultivars showing intermediate spectral responses, suggesting that minor within-group variability in pigment expression contributes to prediction uncertainty. VIP analysis from partial least squares regression independently identified distinct wavelength regions contributing to color discrimination, with peak importance values centered near 500–530 nm and a secondary maximum around 680–720 nm (**Figure 1E**). These regions correspond closely to the wavelengths used in the Δlog(R700 − R520) index, providing quantitative support for the band selection and confirming that visible-range absorption features dominate grain coat color differentiation.

#### 3.1.2 External grain morphology and crease geometry

**Figure 2:**
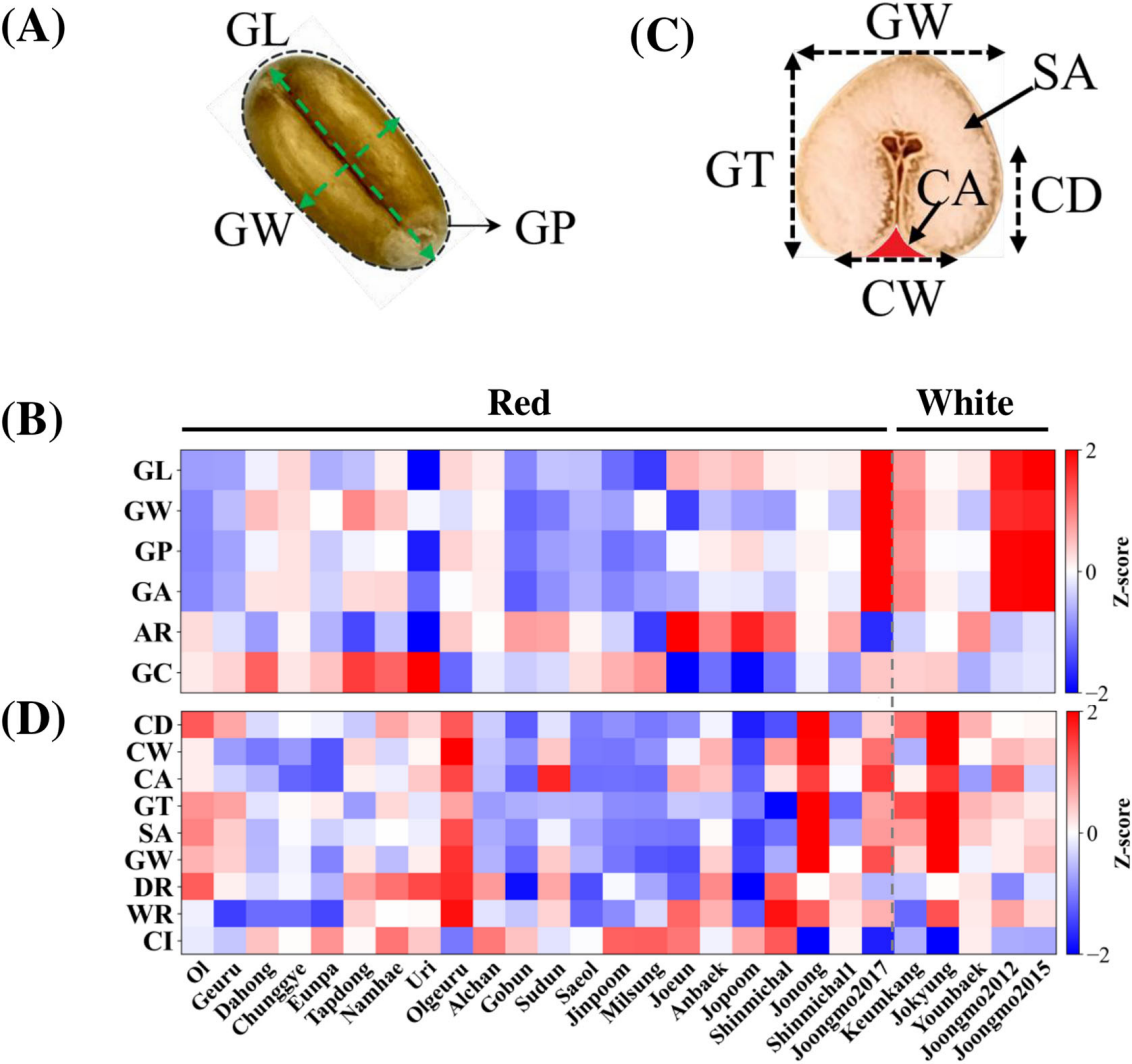
External and internal morphological features of wheat kernels. **(A)** Definition of external grain morphology parameters measured from RGB images of intact kernels, including grain length (GL), grain width (GW), and grain perimeter (GP), from which aspect ratio (AR) and grain circularity (GC) were derived. **(B)** Z-score–normalized external morphological traits for 27 wheat cultivars. Each column represents one cultivar (12 kernels per cultivar), and rows correspond to GL, GW, GP, grain area (GA), AR, and GC. Red and white grain-coat groups are indicated at the top. **(C)** Definition of internal crease geometry parameters measured from high-resolution RGB images of transversely sectioned kernels, including crease depth (CD), crease width (CW), crease area (CA), grain thickness (GT), and grain cross-sectional area (SA). **(D)** Z-score–normalized internal structural and creaseness-related traits for the same 27 cultivars (24 kernels per cultivar). Rows correspond to CD, CW, CA, GT, SA, depth ratio (DR = CD/GT), width ratio (WR = CW/GW), crease area ratio (CR = CA/(SA+CA)), and the composite crease index (CI). Color scale indicates standardized deviation from the mean across cultivars (red, high; blue, low).

External and internal morphological traits of wheat grains were quantified across the cultivar panel. External morphology parameters—grain length (GL), grain width (GW), grain perimeter (GP), grain area (GA), aspect ratio (AR), and grain circularity (GC)—were derived from RGB images generated from hyperspectral data (**Figures 2A, B**). After Z-score normalization, white kernels tended to exhibit higher values for GL, GW, GP, and GA, indicating differences in overall grain size distribution relative to red cultivars. In contrast, AR and GC showed relatively limited variation among white kernels, whereas red kernels exhibited broader distributions, indicating greater shape diversity in grain shape.

Internal structural features related to crease geometry were quantified from RGB images of transversely sectioned kernels (**Figure 2C**). These included absolute measures of crease depth (CD) (Song et al., 2023; Strange et al., 2015), crease width (CW), and crease area (CA), as well as grain thickness (GT) and cross-sectional area (SA). To provide a scale-independent representation of creaseness, dimensionless indices were calculated, including depth ratio (DR = CD/GT), width ratio (WR = CW/GW), and crease area ratio (CR = CA/(SA + CA)). Among these, the composite crease index (CI), defined as CI = (CD/GT) × (CW/GW^2^), was used as the primary descriptor of creaseness, as it integrates both vertical penetration and lateral extent of the crease into a single metric. Other indices (DR, WR, CR) were used as complementary descriptors to capture specific geometric aspects of crease structure. At the cultivar level, crease-related parameters showed no clear separation between red and white grain types following normalization (**Figure 2D**), indicating that creaseness, as defined by these geometric indices, is largely independent of grain coat color. For visualization purposes, kernels were grouped into relative creaseness classes based on CI; however, these classes were not used for statistical analysis, and creaseness was treated as a continuous trait throughout this study.

Coefficient of variation (CV) analysis revealed that crease-related traits such as CA and CR, as well as kernel hardness, exhibited relatively high variability (CV ≈ 17–30%), whereas external morphology traits (e.g., GL, GC) and normalized geometric indices (e.g., DR, CI) showed lower variability (CV < 10%) (**Supplementary Table S4**).

#### 3.1.3 Kernel hardness and protein-starch composition

**Figure 3:**
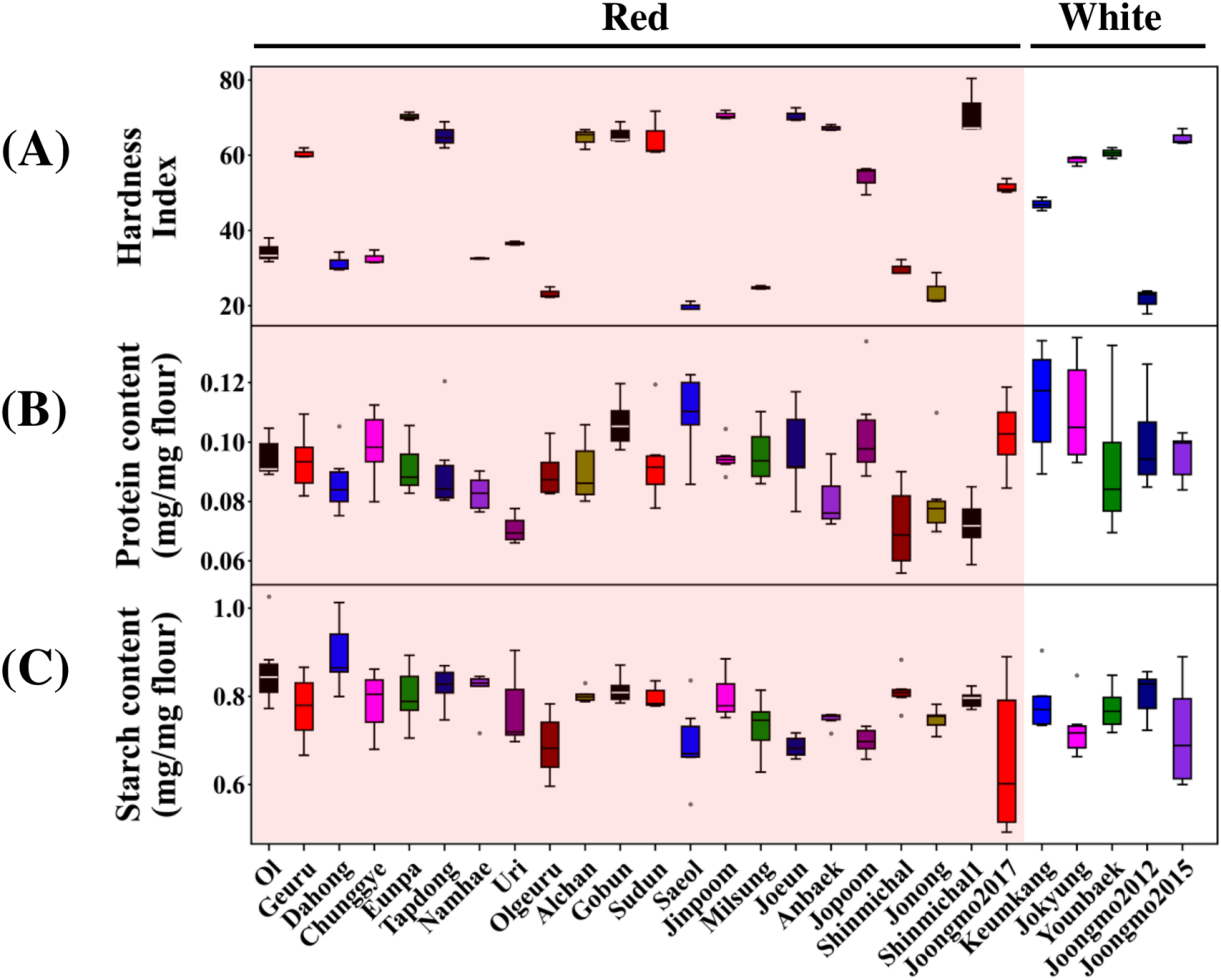
Kernel hardness and biochemical composition of wheat cultivars. **(A)** Kernel hardness measured using the Single Kernel Characterization System (SKCS). Values represent the mean hardness index calculated from 300 individual kernels per cultivar (n = 3). **(B)** Protein content, expressed as mg protein per mg flour, measured from six individual kernels per cultivar. **(C)** Starch-derived glucose content, expressed as mg per mg flour, measured from an independent set of six kernels per cultivar. In (**A–C**), cultivars are ordered consistently and grouped by grain coat color (red and white). Boxplots show median, interquartile range, and individual kernel values.

KH and biochemical composition were evaluated across the cultivar panel. KH, reflecting starch-protein adhesion within endosperms (Pasha et al., 2010), was measured using SKCS. Consistent with previous reports on Korean wheat cultivars (Park et al., 2021), KH values spanned a wide range across cultivars and formed two broad groups corresponding to softer and harder kernels, with several intermediate cases (**Figure 3A**). This variation showed no clear separation by grain coat color. Kernel protein content ranged from approximately 7.0% to 12.5%, while starch content ranged from 60% to 90% across cultivars (**Figures 3B, C**). Protein and starch contents did not cluster according to grain coat color, indicating that these chemical compositional traits are independent of kernel pigmentation.

Coefficient of variation (CV) analysis revealed that protein content displayed higher variabilitythan starch (**Supplementary Table S4**), indicating that compositional variation is unevenly distributed across traits. These quantitative patterns support the observed differences in variability and trait distribution described above.

### 3.2 Variability of vitreousness assessed by spectral unmixing

**Figure 4:**
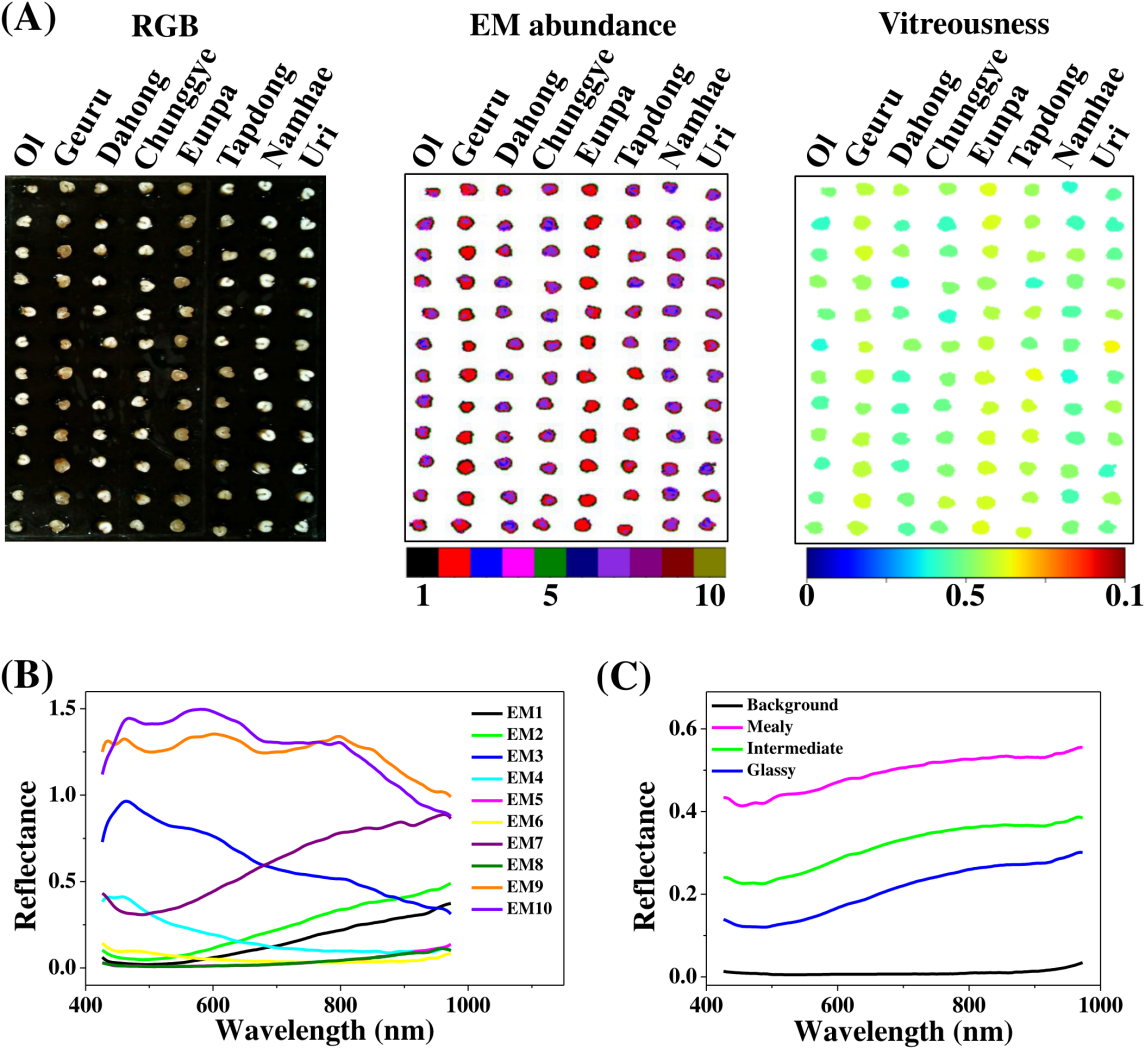
Hyperspectral unmixing and vitreousness mapping workflow. **(A)** RGB image of wheat kernels arranged for hyperspectral acquisition, corresponding pixel-wise endmember abundance maps obtained by linear spectral unmixing, and the resulting kernel-level vitreousness index map, illustrating spatial heterogeneity within and among kernels. **(B)** Spectral signatures of ten endmembers extracted from the hyperspectral cube using the Pixel Purity Index (PPI) algorithm, representing dominant spectral components of endosperm and background. **(C)** Mean reflectance spectra of pixels classified as mealy, intermediate, and glassy endosperm, together with background pixels, showing systematic differences in visible and near-infrared reflectance associated with endosperm structure.

Vitreousness was quantified in a total of 27 Korean wheat cultivars using custom Python-basedhyperspectral analysis (**Supplementary Code S2**). For each acquisition, kernels from 8 cultivars were arranged in a 12 × 8 matrix on a black, low-reflectance platform (**Figure 4A**, RGB). Hyperspectral images were acquired in five batches, including three images of 12 × 8 kernels and one image of 12 × 3 kernels. These images were subjected to spectral unmixing on a per-kernel basis. Initial unmixing yielded ten endmembers, which were used to remove background, shadow, and noise pixels. Kernel regions of interest were then isolated, and spectral unmixing was repeated to obtain kernel-specific endmembers (**Figure 4B**). The resulting endmembers clustered into three spectral types: low-reflectance flat spectra (EM5, EM6, EM8), visible-dominant spectra (EM3, EM4, EM9, EM10), and NIR-dominant spectra (EM1, EM2, EM7).

Pixel-level abundance maps enabled spatial separation of grain coat, glassy, intermediate, and mealy endosperm regions within individual kernels (**Figure 4A**, EM abundance). Mean reflectance spectra extracted from these regions showed that mealy areas had the highest reflectance, glassy regions the lowest, and background pixels negligible reflectance (**Figure 4C**), consistent with differences in endosperm packing density and porosity. Based on their spectral signatures and spatial co-localization with visually defined tissue types, EM1 was associated primarily with glassy endosperm, EM7 with intermediate endosperm, and EM9 with mealy endosperm. An example of the classification workflow linking endosperm structure, spectral signatures, and abundance-based mapping is shown in **Supplementary Figure S2**.

**Figure 5:**
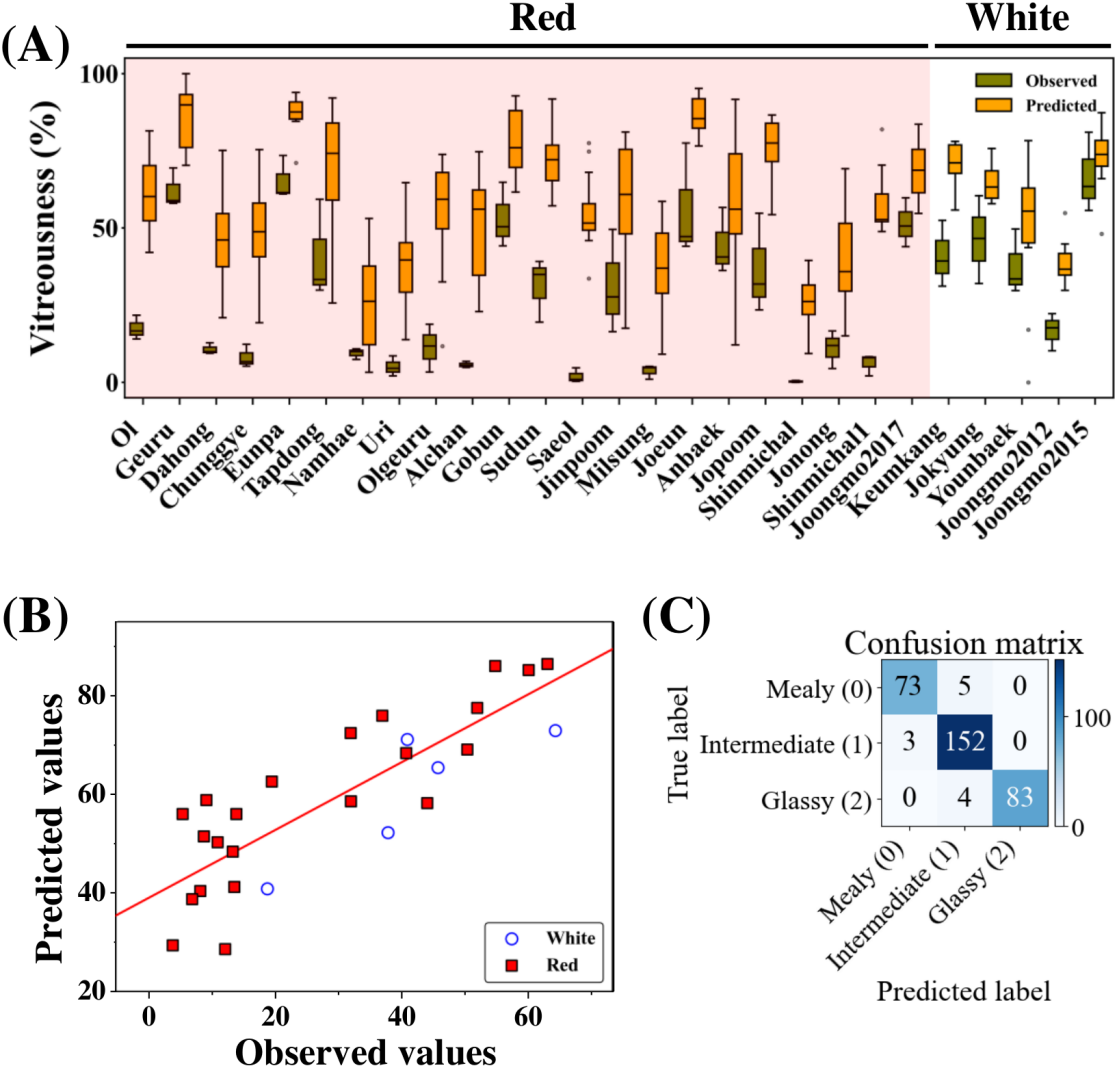
Validation of hyperspectral unmixing–based vitreousness assessment. **(A)** Comparison of vitreousness distributions obtained by visual inspection and hyperspectral spectral unmixing. Visual vitreousness was assessed from transversely sectioned kernels, whereas hyperspectral vitreousness was derived from pixel-level abundance maps. **(B)** Cultivar-level validation of hyperspectral vitreousness, shown as the relationship between visually assessed and hyperspectral vitreousness indices. Each point represents the mean value for one cultivar. The red line indicates linear regression. Model performance for the overall regression is R^2^ = 0.70 and RMSE = 32.04. Group-specific statistics are as follows: red cultivars (n = 22, RMSE = 34.2) and white cultivars (n = 5, RMSE = 19.6). **(C)** Kernel-level validation of the prediction pipeline. Confusion matrix comparing Random Forest–predicted vitreousness classes (mealy, intermediate, glassy), derived from hyperspectral features, with visually assigned classes based on RGB images reconstructed from hyperspectral data.

To validate the script-based vitreousness classification, hyperspectral estimates were compared with visually assigned reference scores. A kernel-level vitreousness index (V) was calculated from the abundance of glassy and intermediate endmembers derived from spectral unmixing and subsequently aggregated to obtain cultivar level vitreousness values (**Figure 5A**). These estimates were compared with vitreousness scores obtained by visual inspection of transversely sectioned kernels. Visual scoring exhibited greater within-cultivar variation than hyperspectral estimates, whereas cultivar-mean values derived from the two approaches showed a strong positive correlation (**Figure 5B**; R^2^ = 0.70, 95% CI: 0.44 – 0.85). When stratified by grain coat color, white cultivars exhibited lower RMSE values compared to red cultivars; however, this difference reflects a narrower range of vitreousness values within the white group rather than systematic differences in model performance.

At the individual kernel level, Random Forest classification based on hyperspectral endmember features achieved high classification performance when evaluated against independently assigned visual classes derived from RGB images reconstructed from hyperspectral cubes (**Figure 5C**). Misclassification was primarily confined to adjacent classes, with minimal confusion between mealy and fully glassy kernels.

### 3.3 Vitreousness and biochemical composition

**Figure 6:**
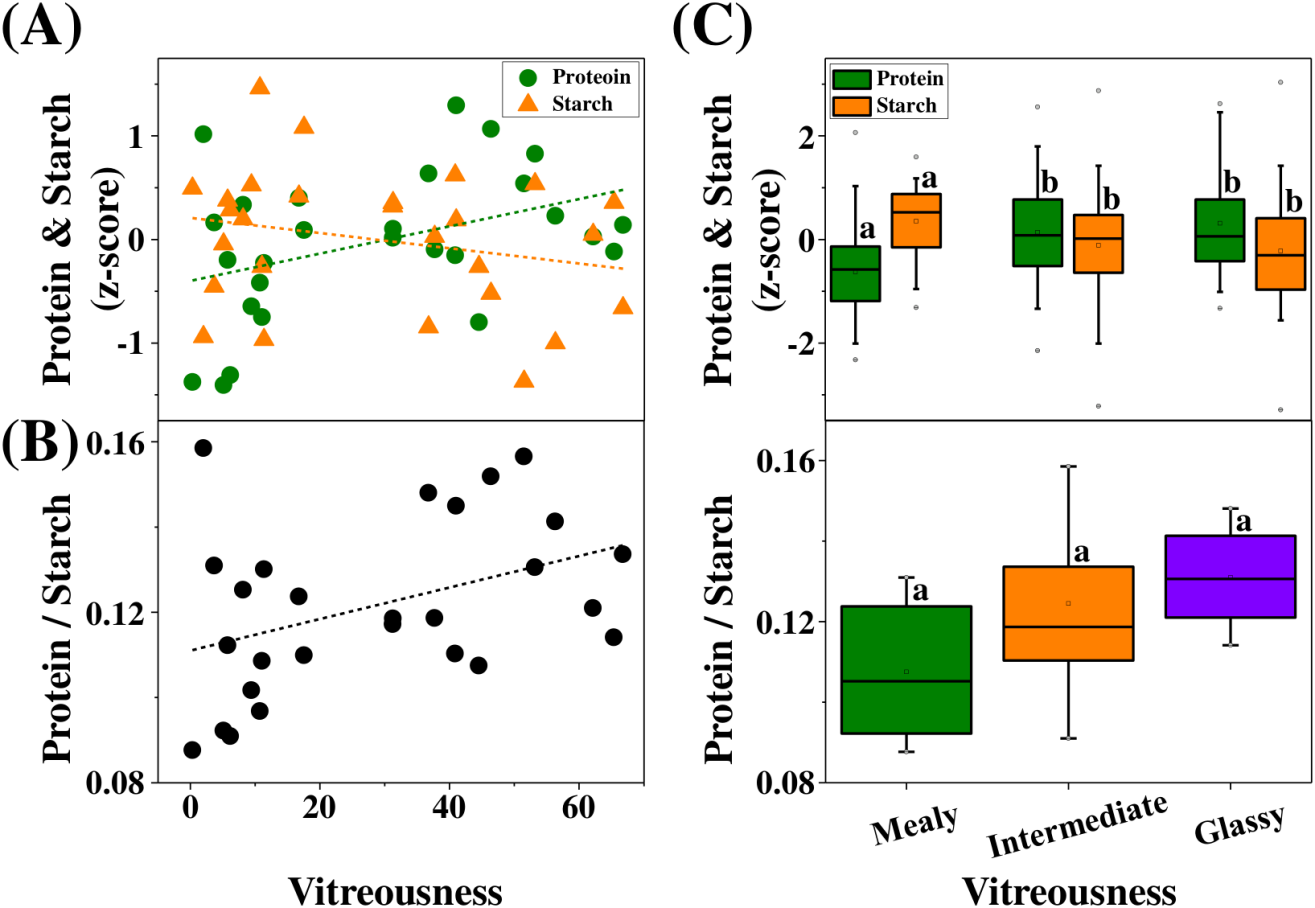
Associations between hyperspectral vitreousness and kernel biochemical composition. (**A–B**) Relationships between cultivar-mean vitreousness index and kernel biochemical traits. **(A)** Protein content (green circles) and starch content (orange triangles), expressed as Z-scores. **(B)** Protein-to-starch ratio. Dashed lines indicate linear regressions, with model performance summarized as follows: protein (R^2^ = 0.17, 95% CI: 0.00 – 0.46; RMSE = 0.63), starch (R^2^ = 0.06, 95% CI: 0.00 – 0.32; RMSE = 0.64), and protein-to-starch ratio (R^2^ = 0.17, 95% CI: 0.00 – 0.47; RMSE = 0.01). **(C)** Distribution of kernel protein and starch contents, and protein-to-starch ratio across vitreousness classes (mealy, intermediate, glassy). Different letters indicate significant differences among groups (p < 0.05, one-way ANOVA). Effect sizes are reported as η^2^ (protein = 0.14, starch = 0.05, protein-to-starch ratio = 0.17). Sample sizes differed among variables: for protein, n = 42 (mealy), 75 (intermediate), and 51 (glassy); for starch, n = 49 (mealy), 82 (intermediate), and 37 (glassy); and for the protein-to-starch ratio, n = 6 (mealy), 17 (intermediate), and 5 (glassy).

Kernel protein content showed a positive association with vitreousness, whereas starch content exhibited a weak negative relationship (**Figure 6A**). At the cultivar level, these relationships were modest (protein R^2^ = 0.17, 95% CI: 0.00 – 0.46; starch R^2^ = 0.06, 95% CI: 0.00 – 0.32). The protein-to-starch ratio increased with vitreousness (**Figure 6B**; R^2^ = 0.17, 95% CI: 0.00 – 0.47). When kernels were grouped into vitreousness classes, protein content differed significantly among classes (p < 0.001, η^2^ = 0.14), whereas starch content showed weaker separation despite a significant overall effect (p = 0.011, η^2^ = 0.05) (**Figure 6C**). The protein-to-starch ratio showed an increasing trend from mealy to glassy kernels, but differences among classes were not statistically significant (**Figure 6C**; p = 0.097, η^2^ = 0.17). The relatively low coefficients are consistent across cultivars and likely reflect underlying biological variability in endosperm structure rather than measurement noise alone.

### 3.4 Creaseness, KH, vitreousness, and biochemical composition

**Figure 7:**
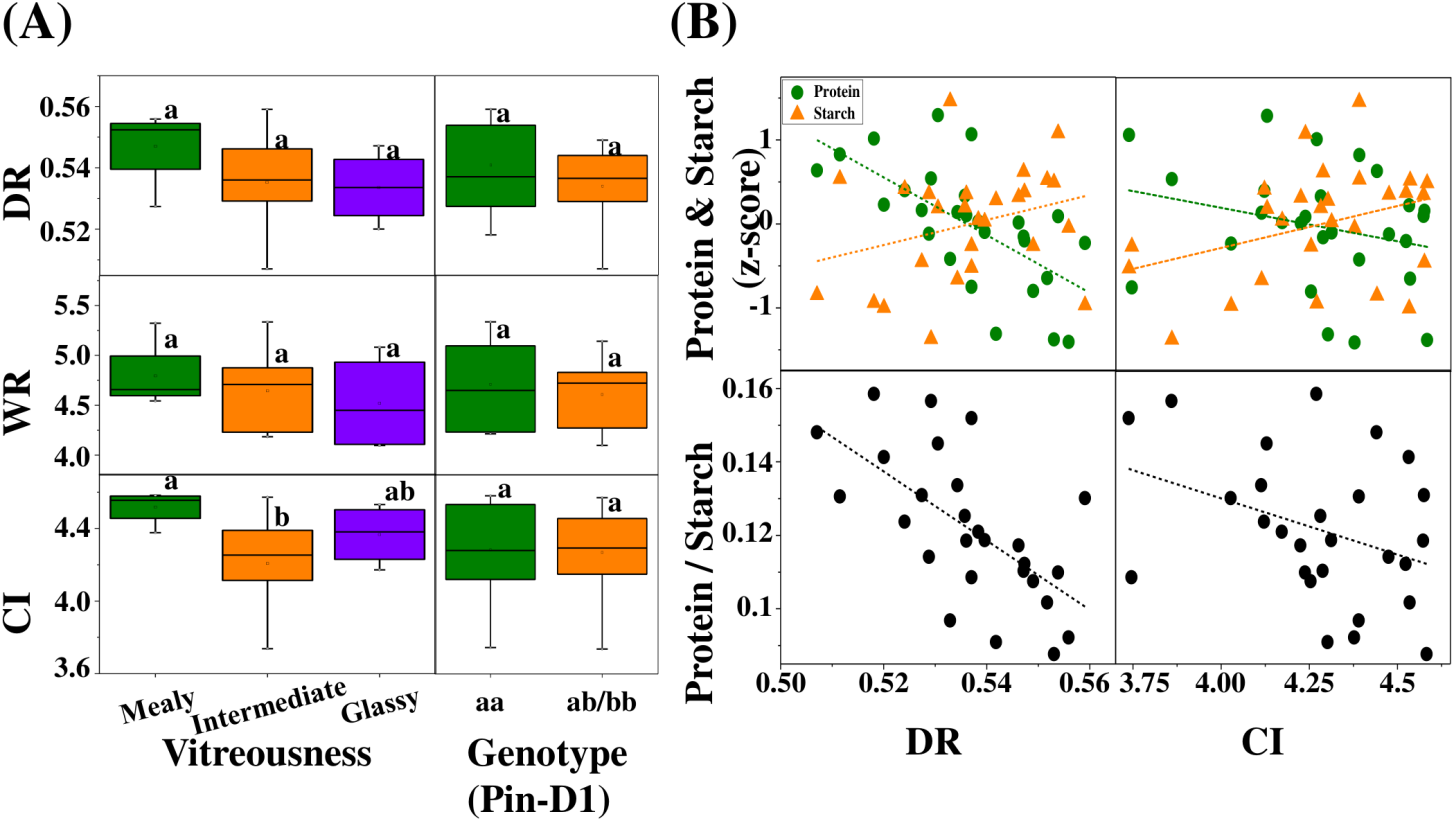
Crease geometry in relation to vitreousness, puroindoline genotype, and kernel composition. **(A)** Distributions of crease depth ratio (DR = CD/GT), width ratio (WR = CW/GW), and composite crease index (CI = CD/GT × CW/GW^2^) across vitreousness classes (mealy, intermediate, glassy; left) and puroindoline genotypes (Pin-D1_aa and Pin-D1_ab; right). Different letters indicate significant differences among groups (p < 0.05, one-way ANOVA). Effect sizes are reported as η^2^ (DR = 0.11, WR = 0.04, CI = 0.24). For vitreousness classes, group sizes were n = 4 (mealy), n = 19 (intermediate), and n = 4 (glassy). For puroindoline genotypes, group sizes were n = 11 (Pin-D1_aa) and n = 14 (Pin-D1_ab/bb). **(B)** Relationships between crease geometry indices (DR and CI) and kernel biochemical traits at the cultivar level. Scatter plots show protein content (Z-score), starch content (Z-score), and protein-to-starch ratio plotted against DR (left) and CI (right). Dashed lines indicate linear regressions, with model performance summarized as follows: For DR: protein (R^2^ = 0.42, 95% CI: 0.13 – 0.68; RMSE = 0.53), starch (R^2^ = 0.09, 95% CI: 0.00 – 0.37; RMSE = 0.63), and protein-to-starch ratio (R^2^ = 0.41, 95% CI: 0.11 – 0.67; RMSE = 0.02). For CI: protein (R^2^ = 0.07, 95% CI: 0.00 – 0.34; RMSE = 0.67), starch (R^2^ = 0.12, 95% CI: 0.00 –0.42; RMSE = 0.62), and protein-to-starch ratio (R^2^ = 0.13, 95% CI: 0.00 – 0.43; RMSE = 0.02).

**Figure 8:**
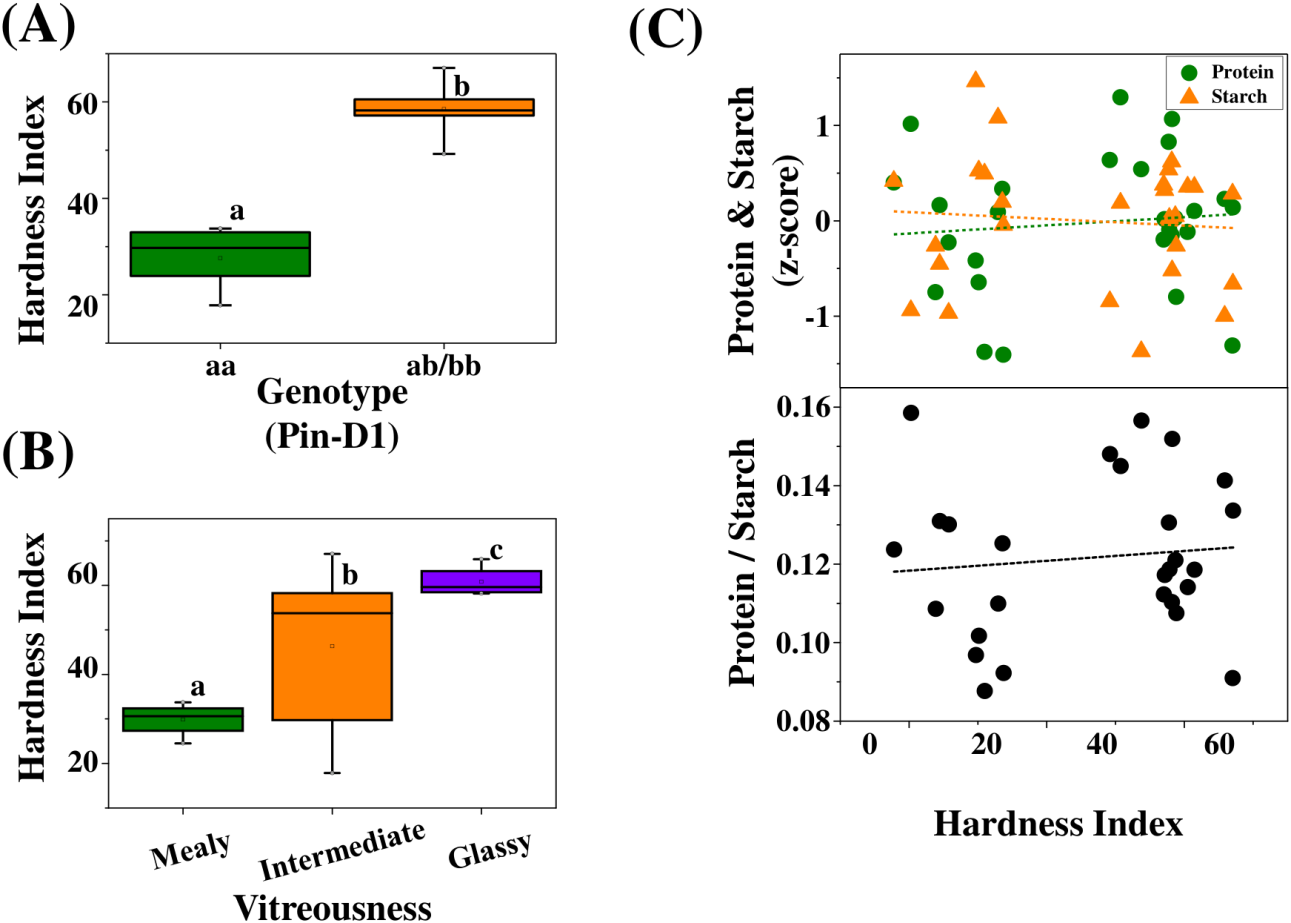
Genetic control of kernel hardness and its relationship with vitreousness and composition. **(A)** Distribution of kernel hardness (SKCS index) for cultivars carrying wild-type (Pin-D1_aa) and mutant (Pin-D1_ab) puroindoline alleles. Group sizes were n = 11 (Pin-D1_aa) and n = 16 (Pin-D1_ab). Differences were significant (p < 0.001, one-way ANOVA; η^2^ = 0.88). **(B)** Distribution of kernel hardness across vitreousness classes (mealy, intermediate, glassy). Group sizes were n = 4 (mealy), n = 19 (intermediate), and n = 4 (glassy). Differences were significant (p = 0.018, one-way ANOVA; η^2^ = 0.28). **(C)** Relationships between kernel hardness and protein content (Z-score), starch content (Z-score), and protein-to-starch ratio at the cultivar level. Dashed lines indicate linear regressions, with model performance summarized as follows: protein (R^2^ = 0.02, 95% CI: 0.00 – 0.18; RMSE = 0.70), starch (R^2^ = 0.00, 95% CI: 0.00 – 0.15; RMSE = 0.66), and protein-to-starch ratio (R^2^ = 0.01, 95% CI: 0.00 – 0.16; RMSE = 0.02).

Crease geometry was quantified using normalized and composite indices derived from high-resolution cross-sectional RGB images, including depth ratio (DR = CD/GT), width ratio (WR = CW/GW), and a composite crease index (CI = CD/GT × CW/GW^2^). When stratified by vitreousness class, DR (*p* = 0.259, η^2^ = 0.11) and WR (*p* = 0.583, η^2^ = 0.04) did not differ significantly among mealy, intermediate, and glassy kernels (**Figure 7A**). In contrast, CI differed significantly among vitreousness classes (*p* = 0.039, η^2^ = 0.24), with mealy kernels exhibiting higher CI values than intermediate and glassy kernels. None of the crease indices differed significantly between puroindoline-defined hardness groups (**Figure 7A**), indicating that crease geometry is independent of kernel hardness. Correlation analyses showed that crease depth ratio (DR) was weakly associated with protein content (R^2^ = 0.42, 95% CI: 0.13 – 0.68) and the protein-to-starch ratio (R^2^ = 0.41, 95% CI: 0.11 – 0.67), whereas its association with starch content was minimal (R^2^ = 0.09, 95% CI: 0.00 – 0.37). Similarly, the composite crease index (CI) exhibited weak associations with protein (R^2^ = 0.07, 95% CI: 0.00 – 0.34), starch (R^2^ = 0.12, 95% CI: 0.00 – 0.42), and the protein-to-starch ratio (R^2^ = 0.13, 95% CI: 0.00 – 0.43). Overall, these relationships indicate limited predictive power of crease geometry for kernel biochemical composition (**Figure 7B**). Kernel hardness exhibited a strong dependence on puroindoline genotype (**Figure 8A**; *p* < 0.001, η^2^ = 0.88). When grouped by vitreousness class, glassy kernels showed higher mean hardness values than mealy kernels, although substantial overlap was observed among classes (**Figure 8B**). In contrast, correlations between hardness and protein content, starch content, or the protein-to-starch ratio were negligible (R^2^ ≤ 0.02, 95% CIs: 0.00 – 0.47; **Figure 8C**). The absence of consistent trends across vitreousness classes indicates that mechanical hardness and endosperm packing are regulated by distinct underlying mechanisms.

## 4 Discussion

Vitreousness is inherently a spatially heterogeneous property of wheat endosperm, yet conventional methods reduce it to a visual class or kernel-averaged reflectance (Zhao et al., 2023; Özdoğan and Gowen, 2025). By combining hyperspectral imaging with spectral unmixing, this study resolves the spatial distribution of glassy, intermediate, and mealy endosperm within individual kernels, enabling vitreousness to be expressed as a continuous index rather than a categorical score. The close correspondence between hyperspectral-derived indices and visual reference assessments, together with strong classification performance, indicates that spectral unmixing provides an objective and spatially resolved alternative to conventional scoring methods. Although the computational framework shares similarities with previous applications in soybean seed phenotyping (Jeong et al., 2024), the biological problem addressed here is fundamentally different. In soybean, spectral unmixing resolves spatially distinct tissue types with stable spectral identities, whereas wheat vitreousness represents a continuous gradient of packing states within a single tissue. This distinction necessitates problem-specific endmember definitions and interpretation, highlighting the adaptability of spectral unmixing as a platform for structurally distinct phenotyping tasks.

In addition to endosperm-related spectral features, the Δlog(R700 − R520) index provides a complementary descriptor of grain coat characteristics by capturing differences between pigment-sensitive and structure-dominated spectral regions. The 520 nm region reflects visible absorption associated with pigment composition, whereas the 700 nm region is influenced more by scattering and internal structure with reduced pigment interference. As a result, this index integrates compositional and structural information, enabling robust discrimination of grain coat color. In this study, its consistency with hyperspectral classification and PLSR-derived VIP features supports its utility as a compact spectral indicator of grain coat variation.

The modest correlations observed between vitreousness and biochemical composition (R^2^ ≤ 0.17) reflect a fundamental property of the trait rather than a limitation of the analysis. Vitreousness arises from the spatial organization and packing density of the protein–starch matrix within the endosperm, rather than from absolute protein or starch content alone (Baasandorj et al., 2015; Dexter and Edwards, 1998; El‐Khayat et al., 2003; Samson et al., 2005; Sieber et al., 2015). Consequently, kernels with similar bulk composition can exhibit markedly different internal structures depending on how protein is distributed during grain filling. This explains why bulk compositional analyses show limited predictive power, whereas hyperspectral spectral unmixing captures structural variation by resolving differences in endosperm packing at the pixel level.

High-resolution RGB imaging further revealed that crease geometry captures macroscopic structural features of wheat kernels that are largely distinct from endosperm packing density and mechanical hardness. Simple linear descriptors such as crease depth or width alone (Ajiboye et al., 2015; Kong and Baik, 2016; Mabille and Abecassis, 2003; Samson et al., 2005) provided limited sensitivity to variation in endosperm state, whereas composite indices integrating normalized depth and width more effectively reflected differences associated with vitreousness. This indicates that the functional relevance of the ventral groove cannot be adequately represented by a single geometric dimension. Instead, crease geometry appears to encode developmental constraints imposed during grain filling that influence overall kernel morphology rather than directly determining internal packing or molecular adhesion. Because crease geometry can be quantified from standard RGB images without specialized instrumentation, it offers a low-cost, complementary structural descriptor that can be integrated with hyperspectral vitreousness mapping in multi-scale kernel phenotyping.

KH, vitreousness, and crease geometry together represent complementary but largely independent physical dimensions of grain quality. Mechanical hardness is primarily governed by puroindoline genotype (Huertas-García et al., 2023; Tu and Li, 2020) and reflects molecular-scale starch-protein adhesion at the granule surface (Bhave and Morris, 2008). In contrast, vitreousness captures mesoscale endosperm packing density and porosity shaped by protein–starch balance during grain filling (Legland et al., 2023; Turnbull et al., 2003; Yamazaki and Donelson, 1983), while creaseness reflects macroscopic kernel geometry established by developmental patterning of the ventral groove (Ajiboye et al., 2015; Kong and Baik, 2016; Sun et al., 2007). The partial overlap between hardness and vitreousness, combined with their weak associations with bulk composition and crease geometry, underscores that no single trait adequately describes milling-relevant kernel quality. This multi-dimensionality explains why kernels with similar hardness values can differ substantially in internal structure and processing behavior. From a food quality perspective, these findings imply that grain classification systems relying solely on hardness or bulk protein content may systematically misassign kernels with divergent endosperm packing states, potentially leading to inconsistent flour functionality, variable water absorption, and unpredictable dough rheology in downstream processing. Integrating spatially resolved vitreousness indices alongside conventional hardness and protein measurements therefore offers a more complete predictor of milling yield and flour quality relevant to bread, noodle, and flat bread production.

By decoupling mechanical resistance, packing density, and grain geometry, the integrated imaging framework developed here enables discrimination of kernels that would be indistinguishable using conventional single-trait metrics. The ability to simultaneously resolve external grain characteristics and internal endosperm organization supports its application in integrated phenotyping, sorting, and quality-control workflows. More broadly, this approach provides a framework for linking genetic and environmental drivers of grain development to spatially resolved physical traits that directly influence milling performance. From a practical standpoint, the pipeline developed is fully script-driven, requires no proprietary software, and can be implemented using standard VNIR hyperspectral imaging systems. Its non-destructive nature allows kernels to be further used for downstream analysis including milling trials and seed propagation, which is particularly valuable for breeding applications.

Despite these advances, several limitations should be considered. First, vitreousness does not have a direct physical ground truth, and both visual and hyperspectral approaches rely on optical manifestations of internal structure. Second, spectral unmixing requires assumptions regarding endmember selection and class definitions, which may influence quantitative outcomes, although our robustness analyses indicate that overall trends remain stable. Third, the present study was conducted under controlled conditions using a limited cultivar panel, and further validation across diverse genetic backgrounds and environmental conditions is necessary. Future work should integrate hyperspectral imaging with complementary structural techniques, such as X-ray micro-computed tomography, to directly link spectral signatures with three-dimensional endosperm architecture. Extending the framework to fully continuous abundance-based formulations and large-scale breeding populations will further enhance its utility. Together, these results establish hyperspectral spectral unmixing as a scalable and physically grounded approach for quantifying endosperm structure and linking composition, spatial organization, and grain quality.

## 5 Conclusions

This study demonstrates that hyperspectral spectral unmixing enables spatially resolved, non-destructive quantification of wheat kernel vitreousness, capturing internal structural heterogeneity that cannot be resolved by conventional visual or kernel-averaged spectral methods. By integrating hyperspectral analysis with RGB-based crease geometry and mechanical hardness measurements, the proposed framework provides a multi-scale characterization of kernel quality traits. This approach offers a scalable and physically interpretable platform for digital phenotyping in wheat breeding and post-harvest quality assessment.

## Data Availability

All scripts, processed data supporting the findings of this study, including derived feature tables and representative supplementary material are provided in **Supplementary Materials**. Due to their large file size, full hyperspectral image cubes and associated RGB imagedatasets are available via **Research Datas 1**–**3** at Figshare: https://doi.org/10.6084/m9.figshare.31259530.

## Supplementary material

Supplementary Table S1 Comparison of spectral endmember extraction algorithms and reconstruction performance for hyperspectral wheat kernel data.

Supplementary Table S2 Summary of kernel counts used for hyperspectral vitreousness and creaseness analyses across wheat cultivars.

Supplementary Table S3 Processed kernel-level and cultivar-level feature tables used for statistical analyses and figure generation, including measurements of vitreousness, creaseness, kernel hardness, protein content, and starch content.

Supplementary Table S4 Summary statistics and coefficient of variation (CV) for morphological and crease-related traits across wheat kernels.

Supplementary Figure S1 Workflow for high-resolution RGB-based creaseness analysis and phenotyping of wheat kernels.

Supplementary Figure S2 Schematic overview of the hyperspectral imaging and spectral unmixing pipeline for spatially resolved vitreousness assessment in wheat kernels.

Supplementary Code S1 Python scripts for RGB-based creaseness analysis. This file contains scripts for RGB image preprocessing, kernel segmentation, crease detection, and extraction of geometric features used for creaseness quantification.

Supplementary Code S2 Python scripts for hyperspectral imaging (HSI)–based vitreousness analysis. This file contains scripts for hyperspectral data preprocessing, endmember extraction, linear spectral unmixing (LMM/NNLS), and computation of the kernel-level vitreousness index.

Research Data 1 RGB images derived from hyperspectral cubes of intact kernels and kernel cross-sections for spectral unmixing.

Research Data 2 RGB camera images of kernel cross-sections used for visual vitreousness assessment.

Research Data 3 High-resolution RGB camera images of kernel cross-sections used for creaseness assessment.
