# Supplementary material for: Spatially resolved quantification of wheat kernel vitreousness using hyperspectral imaging and spectral unmixing
Source: Front Plant Sci. 2026 May 18;17:1832288. doi: 10.3389/fpls.2026.1832288 (PMC13222845; doi:10.3389/fpls.2026.1832288)
Supplement: Supplementary Table S1 — Comparison of spectral endmember extraction algorithms and reconstruction performance for hyperspectral wheat kernel data. [file Table1.docx]

**Supplementary Table S1. Comparison of spectral endmember extraction algorithms and reconstruction performance for hyperspectral wheat kernel data.**

Performance of three endmember extraction algorithms—Pixel Purity Index (PPI), N-FINDR, and Fast Iterative Pixel Purity Index (FIPPI)—is summarized based on hyperspectral datasets comprising 4 hypercubes and 324 kernel images. For each method, the optimal number of endmembers (EMs) and reconstruction accuracy are reported. Reconstruction performance was evaluated using relative root mean square error (rRMSE), expressed as mean ± standard deviation across hypercubes. Lower rRMSE values indicate better reconstruction of observed spectral signals. These results demonstrate that the spectral unmixing framework yields comparable performance across different endmember extraction methods, supporting the robustness of the derived vitreousness estimates.

| Algorithm | Hypercubes | Images (Number of seeds) | Optimal EMs | rRMSE |
| --- | --- | --- | --- | --- |
| PPI | 4 | 324 | 10 | 0.1346 ± 0.0126 |
| N-FINDR | 4 | 324 | 13 | 0.1548 ± 0.0211 |
| FIPPI | 4 | 324 | 12 | 0.1986 ± 0.0192 |
