# Supplementary material for: Spatially resolved quantification of wheat kernel vitreousness using hyperspectral imaging and spectral unmixing
Source: Front Plant Sci. 2026 May 18;17:1832288. doi: 10.3389/fpls.2026.1832288 (PMC13222845; doi:10.3389/fpls.2026.1832288)
Supplement: Supplementary Table S2 — Summary of kernel counts used for hyperspectral vitreousness and creaseness analyses across wheat cultivars. [file Table2.docx]

**Supplementary Table S2. Summary of kernel counts used for hyperspectral vitreousness and creaseness analyses across wheat cultivars.**

For each cultivar, the number of kernels included in the hyperspectral imaging–based vitreousness analysis (“Vitreousness Set”) and the independent kernel subset used for high-resolution RGB-based creaseness analysis (“Creaseness Set”) are reported. The vitreousness dataset consisted of 126 kernels per cultivar, acquired across multiple imaging batches (12 × 8 kernel layout per image), ensuring balanced representation and minimizing batch effects. The creaseness dataset was derived from an independent subset of kernels (n = 16–25 per cultivar), imaged separately under high-resolution RGB conditions due to spatial resolution constraints of the hyperspectral system. These datasets were treated as independent biological replicates for each phenotyping modality, and were integrated at the cultivar level for statistical analyses.

| Group | Vitreousness Set | Creaseness Set | |
| --- | --- | --- | --- |
| Ol | 126 | 21 |  |
| Geuru | 126 | 16 |  |
| Dahong | 126 | 22 |  |
| Chunggye | 126 | 24 |  |
| Eunpa | 126 | 20 |  |
| Tapdong | 126 | 24 |  |
| Namhae | 126 | 24 |  |
| Uri | 126 | 22 |  |
| Olgeuru | 126 | 24 |  |
| Alchan | 126 | 24 |  |
| Gobun | 126 | 23 |  |
| Keumkang | 126 | 22 |  |
| Seodun | 126 | 22 |  |
| Saeol | 126 | 22 |  |
| Jinpoom | 126 | 23 |  |
| Milsung | 126 | 23 |  |
| Joeun | 126 | 20 |  |
| Anbaek | 126 | 23 |  |
| Jopum | 126 | 19 |  |
| Shinmichal | 126 | 24 |  |
| Jonong | 126 | 25 |  |
| Jokyung | 126 | 25 |  |
| Younbaek | 126 | 24 |  |
| Shinmichal1 | 126 | 23 |  |
| Joongmo2012 | 126 | 23 |  |
| Joongmo2015 | 126 | 24 |  |
| Joongmo2017 | 126 | 22 |  |
