# Supplementary material for: Spatially resolved quantification of wheat kernel vitreousness using hyperspectral imaging and spectral unmixing
Source: Front Plant Sci. 2026 May 18;17:1832288. doi: 10.3389/fpls.2026.1832288 (PMC13222845; doi:10.3389/fpls.2026.1832288)
Supplement: Supplementary Table S4 — Summary statistics and coefficient of variation (CV) for morphological and crease-related traits across wheat kernels. [file Table4.docx]

**Supplementary Table S4. Summary statistics and coefficient of variation (CV) for morphological and crease-related traits across wheat kernels.**

Mean, standard deviation (Std_Dev), and coefficient of variation (CV, %) are reported for all measured morphological and creaseness-related parameters, including crease depth (CD; measured using two methods, H and C), crease width (CW), crease area (CA), grain thickness (GT), and cross-sectional area (SA), as well as derived dimensionless indices such as depth ratio (DR), width ratio (WR), crease ratio (CR), and composite crease index (CI). In addition, biochemical traits (protein content, starch content) and kernel hardness index are included to enable comparison between structural and compositional variability. The coefficient of variation (CV) was calculated as (standard deviation / mean) × 100 and is used to quantify relative variability across traits. These quantitative summaries provide an objective basis for evaluating variability patterns described in Sections 3.1.2 and 3.1.3. Overall, structural traits such as crease area (CA), crease ratio (CR), and hardness index exhibit relatively high variability (CV > 15–25%), whereas normalized geometric indices (e.g., DR, CI) and external morphology parameters (e.g., GL, GC) show lower variability (CV < 10%), indicating more stable descriptors across cultivars. Biochemical traits display intermediate variability, with protein showing higher dispersion than starch.

|  | Mean | Std_Dev | CV_Percentage |
| --- | --- | --- | --- |
| GL | 6.542 | 0.2081 | 3.180865 |
| GW | 3.0442 | 0.1247 | 4.09498 |
| GP | 15.674 | 0.5273 | 3.364217 |
| GA | 15.632 | 1.0352 | 6.62248 |
| AR | 2.167 | 0.0782 | 3.607494 |
| GC | 0.7966 | 0.0153 | 1.917644 |
| CD | 605.52 | 37.911 | 6.260805 |
| CW | 585.53 | 76.217 | 13.01674 |
| CA | 34775 | 8482.3 | 24.39201 |
| GT | 1122.8 | 69.229 | 6.165703 |
| SA | 1088008 | 135034 | 12.41107 |
| DR | 0.4821 | 0.0183 | 3.790567 |
| WR | 0.4629 | 0.0378 | 8.174801 |
| CR | 0.0307 | 0.0053 | 17.26678 |
| CI | 4.2765 | 0.2374 | 5.551719 |
| Protein | 0.0928 | 0.0161 | 17.35724 |
| Starch | 0.7698 | 0.0842 | 10.94233 |
| Hardness Index | 42.103 | 12.595 | 29.91354 |
