# Supplementary material for: Spatially resolved quantification of wheat kernel vitreousness using hyperspectral imaging and spectral unmixing
Source: Front Plant Sci. 2026 May 18;17:1832288. doi: 10.3389/fpls.2026.1832288 (PMC13222845; doi:10.3389/fpls.2026.1832288)
Supplement: Supplementary Figure S1 — Workflow for high-resolution RGB-based creaseness analysis and phenotyping of wheat kernels. [file DataSheet1.docx]

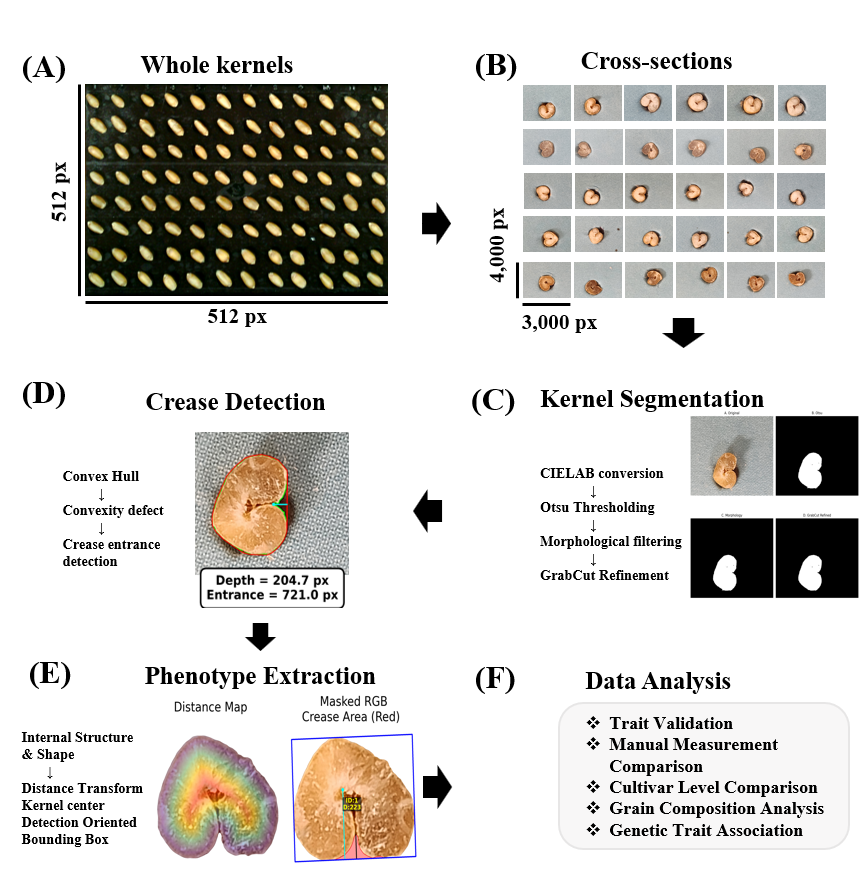


**Supplementary Fig. S1. Workflow for high-resolution RGB-based creaseness analysis and phenotyping of wheat kernels.**

(**A**) Whole kernels arranged for imaging prior to processing (512 × 512 px). (**B**) Transversely sectioned kernels imaged at high resolution (approximately 3,000 × 4,000 px) to capture internal structural features. (**C**) Kernel segmentation pipeline, including CIELAB color space conversion, Otsu thresholding, morphological filtering, and GrabCut refinement to isolate kernel regions from the background. (**D**) Automated crease detection based on convex hull construction and convexity defect analysis, enabling identification of the crease entrance and measurement of geometric parameters such as depth and width. (**E**) Phenotype extraction from segmented kernels, including distance transform–based internal structure mapping and quantification of crease-related traits (e.g., crease area and spatial distribution).
(**F**) Downstream data analysis, including trait validation, comparison with manual measurements, cultivar-level comparisons, grain composition analysis, and association with genetic traits.
