# Supplementary material for: Spatially resolved quantification of wheat kernel vitreousness using hyperspectral imaging and spectral unmixing
Source: Front Plant Sci. 2026 May 18;17:1832288. doi: 10.3389/fpls.2026.1832288 (PMC13222845; doi:10.3389/fpls.2026.1832288)
Supplement: Supplementary Figure S2 — Schematic overview of the hyperspectral imaging and spectral unmixing pipeline for spatially resolved vitreousness assessment in wheat kernels. [file DataSheet2.docx]

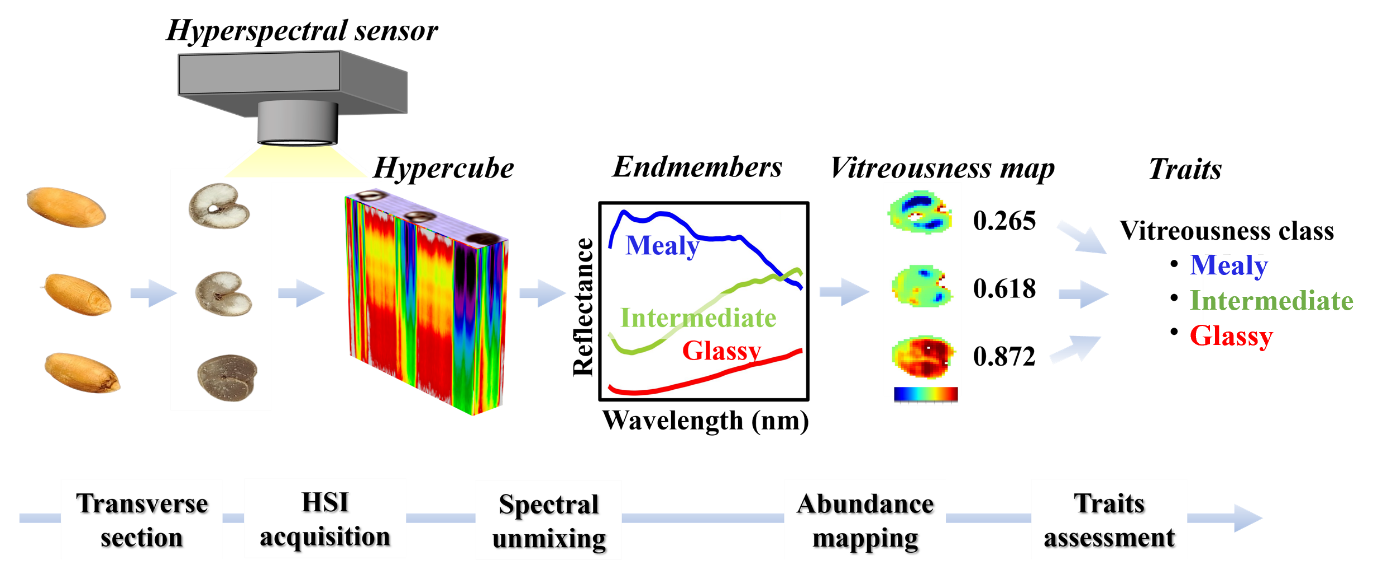


**Supplementary Fig. S2. Schematic overview of the hyperspectral imaging and spectral unmixing pipeline for spatially resolved vitreousness assessment in wheat kernels.**

Wheat kernels were first transversely sectioned to expose internal endosperm structure and imaged using a hyperspectral imaging (HSI) system in the visible–near infrared range. The resulting hyperspectral data cubes were processed using spectral unmixing to decompose each pixel into fractional contributions of representative endmember spectra corresponding to mealy, intermediate, and glassy endosperm components. Pixel-wise abundance maps were then generated to visualize the spatial distribution of these components within individual kernels. Finally, abundance-based segmentation outputs were used to derive quantitative trait measures, including a continuous vitreousness index and associated structural descriptors for downstream analysis.
